# Screening and comprehensive analysis of endoplasmic reticulum stress-related biomarkers in atherosclerosis

**DOI:** 10.1371/journal.pone.0350047

**Published:** 2026-06-01

**Authors:** Xiaomeng Qu, Yiming Shao, Han Li, Yuhan Bao, Zhen Sun, Shuhua Yu

**Affiliations:** 1 Department of Hypertension, Zhengzhou Central Hospital Affiliated to Zhengzhou University, Zhengzhou, Henan, People’s Republic of China; 2 Cell Research and Translational Center, Zhengzhou Central Hospital Affiliated to Zhengzhou University, Zhengzhou, Henan, People’s Republic of China; Versiti Blood Research Institute, UNITED STATES OF AMERICA

## Abstract

Atherosclerosis (AS) is a chronic inflammatory vascular disorder in which endoplasmic reticulum stress (ERS) plays a crucial regulatory role. However, the biological and translational relevance of ERS-related gene networks in AS remains largely unexplored. This study aimed to identify a robust ERS-related gene signature for AS. We integrated multiple GEO datasets and applied machine learning algorithms, including least absolute shrinkage and selection operator (LASSO) regression, support vector machine-recursive feature elimination (SVM-RFE), and random forest (RF). Five ERS-related signature genes (TRIM25, CYBB, CYBA, MYOC, and PRKAA2) were identified and showed favorable discriminatory performance in the integrated discovery cohort (combined AUC = 0.946). The expression patterns of these genes were further examined at both the mRNA and protein levels by quantitative real-time polymerase chain reaction (qRT-PCR) and Western blotting (WB) in an oxidized low-density lipoprotein (ox-LDL)-induced endothelial injury model. Gene set enrichment analysis and immune infiltration analysis indicated that the identified genes were primarily involved in oxidative stress and immune-related pathways. Collectively, this study identifies a machine learning–derived ERS gene signature associated with AS. These findings improve our understanding of ERS-related vascular injury in AS and provide candidate biomarkers for further tissue-level and mechanistic validation.

## 1. Introduction

Atherosclerosis (AS) is a chronic vascular disease that primarily affects large and medium-sized arteries.[1] Progression of atherosclerotic lesions can lead to luminal stenosis or plaque rupture, which constitutes a major mechanism underlying fatal cardiovascular and cerebrovascular events such as acute myocardial infarction and ischemic stroke.[2] As the pathological basis of most atherosclerotic cardiovascular disease (ASCVD), AS imposes a substantial and growing public health burden. Cardiovascular diseases caused an estimated 19.8 million deaths worldwide in 2022, accounting for approximately 32% of all deaths, while in China they remain the leading cause of death and account for nearly half of all deaths [3]; moreover, the number of new cardiovascular disease cases in China increased from approximately 5.3 million in 1990 to 12.3 million in 2019.[4] Therefore, there is an urgent need to identify more precise biomarkers and potential molecular targets for improved risk stratification and mechanistic investigation in AS.

Endoplasmic reticulum stress (ERS) is an adaptive response to the accumulation of unfolded or misfolded proteins in the endoplasmic reticulum (ER).[5] The ER maintains proteostasis by regulating protein folding, lipid synthesis, and calcium homeostasis through molecular chaperones and the ER-associated degradation pathways.[6] When ER function is overwhelmed, the unfolded protein response (UPR) will be activated to restore balance. However, prolonged or excessive stress induces apoptosis and inflammation, accelerating the progression of the disease.[7] Mounting evidence has linked ERS to endothelial dysfunction, foam cell formation, and plaque instability. ERS promotes macrophage apoptosis, contributing to necrotic core enlargement in atherosclerotic plaques.[8] In addition, atherosclerotic risk factors like ox-LDL induce ERS, thereby promoting inflammatory responses and osteogenic differentiation.[9] Similarly, homocysteine (Hcy) has been reported to aggravate atherosclerosis by promoting macrophage pyroptosis through ERS and calcium dysregulation. [10,11] Consistently, activation of the UPR has been observed throughout atherosclerotic lesion development in ApoE-deficient mice.[10] Nevertheless, the association between AS and ERS involves abnormalities across a wide range of genes, proteins, and signaling pathways. Traditional studies focusing on single genes or pathways are insufficient to fully characterize this complexity or identify reliable diagnostic biomarkers.

To address these limitations, this study integrated multiple Gene Expression Omnibus (GEO) datasets and combined bioinformatics with machine learning approaches to systematically identify ERS-related genes in AS. Three complementary machine learning algorithms, including least absolute shrinkage and selection operator (LASSO) regression, support vector machine-recursive feature elimination (SVM-RFE), and random forest (RF) were used to identify robust ERS-related signature genes for distinguishing AS from comparator samples. The expression patterns of these genes were further examined in an oxidized low-density lipoprotein (ox-LDL)-induced endothelial injury model. We also explored their immune-related features. Overall, this study aims to improve the understanding of ERS-related vascular injury in AS and to identify candidate ERS-related markers for further biological and translational investigation.

## 2. Materials and methods

### 2.1. Data acquisition

In this study, three AS-related transcriptome datasets (GSE28829, GSE43292, and GSE100927) were retrieved from the Gene Expression Omnibus (GEO) (https://www.ncbi.nlm.nih.gov/). Datasets were included if they contained human carotid atherosclerosis-related vascular tissue samples with clearly defined comparison groups and adequate sample annotation for reliable lesion-status interpretation. The integrated discovery cohort consisted of GSE28829 (13 early lesions and 16 advanced plaques), GSE43292 (32 intact tissue samples and 32 plaque samples), and GSE100927 (35 control samples and 69 plaque samples). Because the underlying comparison design differed across datasets, comparator groups were defined according to the original dataset annotations rather than being uniformly regarded as normal vascular tissues. Specifically, GSE28829 compared advanced and early atherosclerotic lesions, whereas GSE43292 and GSE100927 compared plaque tissues with intact or control vascular tissues. Overall, the integrated discovery cohort comprised 197 samples, including 117 AS/plaque samples and 80 comparator samples. Public GEO datasets were retrieved in March 2025. All bioinformatics analyses were performed between April 2025 and August 2025. Probe annotation, background correction, and normalization were performed on the raw data using the limma package in R (version 4.4.0). To remove inter-platform batch effects while preserving true biological variation, batch effect correction was conducted using the ComBat function in the sva package (par.prior = TRUE and prior.plots = FALSE) within an empirical Bayes framework. The effectiveness of batch correction was assessed by principal component analysis (PCA), which was visualized using the ggplot2 package. A structured summary of the included datasets, original comparison design, sample composition, and study time frame is provided in Table 1.

**Table 1:** Overview of included GEO datasets and sample composition.

Dataset	Platform	Tissue	Comparator group (n)	AS/ plaque group (n)	AS: Comparator ratio	Role
GSE28829	GPL570	Early vs advanced carotid lesions	13 (early lesions)	16 (advanced plaques)	1.23:1	Discovery
GSE43292	GPL6244	Intact tissue vs plaque tissue	32 (intact tissue)	32 (plaque tissue)	1:1	Discovery
GSE100927	GPL15207	Control vs plaque tissue	35 (control)	69 (plaque tissue)	1.97:1	Discovery
**Overall**	—	Integrated discovery cohort	80	117	1.46:1	Discovery

### 2.2. Acquisition of ERS-related genes

The GeneCards database (https://www.Genecards.org/) was searched with the keyword “endoplasmic reticulum stress” and the genes that had a relevance score ≥10 were screened. The ERS-related gene sets were also searched in the Molecular Signatures Database (MSigDB) (https://www.gsea-msigdb.org/). The merged and deduplicated genes were used for the subsequent analysis.

### 2.3. Differential expression analysis

Differential expression analysis was performed on the integrated, batch-corrected expression matrix using the limma package in R. For each gene, differential expression between the AS and comparator groups was assessed using the empirical Bayes moderated *t*-tests implemented in limma. Raw *P* values were adjusted for multiple testing using the Benjamini–Hochberg method, and genes with | log_2_(Fold Change) | > 0.585 and adjusted *P* < 0.05 were considered differentially expressed genes (DEGs). The AS-related DEGs were then intersected with the ERS gene set using the VennDiagram package to identify ERS-related DEGs. A volcano plot was generated to visualize the global DEG landscape, with the 38 ERS-related DEGs specifically highlighted.

### 2.4. Functional enrichment analysis

Functional enrichment analyses of the 38 ERS-related differentially expressed genes (DEGs) were performed using the clusterProfiler package in R. Gene Ontology (GO) enrichment analysis was conducted for the three GO categories, including biological process (BP), cellular component (CC), and molecular function (MF), to characterize the potential biological functions of these genes. Kyoto Encyclopedia of Genes and Genomes (KEGG) pathway enrichment analysis was further performed to identify significantly enriched signaling pathways. The Benjamini–Hochberg method was used for multiple-testing correction, and terms with adjusted *P* < 0.05 were considered significantly enriched. Enrichment results were visualized as dot plots, with *GeneRatio* shown on the x-axis, dot size representing *Count*, and dot color indicating adjusted *P* value.

To further explore pathways associated with individual gene expression, we performed gene set enrichment analysis (GSEA) using gene expression profiles from the AS dataset. GSEA was performed using the clusterProfiler package in R software with gene sets from the KEGG collection (c2.cp.kegg.Hs.symbols.gmt). Significantly enriched pathways were identified based on *P* < 0.05.

### 2.5. Machine learning-based feature selection and receiver operating characteristic analysis

Three complementary machine learning algorithms were applied to the 38 ERS-related differentially expressed genes to identify robust ERS-related signature genes for distinguishing AS from comparator samples. LASSO regression, SVM-RFE, and RF were selected because they capture complementary aspects of feature importance, thereby minimizing algorithm-specific bias and improving the stability of feature selection. LASSO regression was performed using the cv.glmnet function in the glmnet R package under a binomial generalized linear model, with AS status specified as the binary outcome. Ten-fold cross-validation was used to determine the optimal penalty parameter (λ), and genes with non-zero coefficients were retained. SVM-RFE was performed using the known class labels to identify genes with the strongest discriminative contribution, while RF analysis was used to rank genes according to variable importance. Genes identified by all three algorithms were retained as candidate signature genes. To assess their discriminatory performance in the integrated dataset, receiver operating characteristic (ROC) curves were generated using the pROC R package, and the area under the curve (AUC) was calculated for each individual gene. In addition, a binary logistic regression model based on the expression profiles of the five selected genes was constructed, and its overall discriminatory performance was evaluated by ROC analysis. The corresponding AUC and 95% confidence interval (95% CI) were calculated.

### 2.6. CIBERSORT immune cell infiltration analysis

The proportions of 22 immune cell subtypes in the AS dataset were quantified using the CIBERSORT algorithm (https://cibersort.stanford.edu/). Spearman correlation analysis was performed between signature gene expression and the CIBERSORT-estimated fractions of 22 immune cell types across samples, and *P* < 0.05 was considered statistically significant.

### 2.7. Cell culture and viability assay

Human umbilical vein endothelial cells (HUVECs) were purchased from Procell (China) and cultured at 37℃ with 5% CO_2_ in endothelial cell medium (ScienCell, USA). Cell viability was assessed using the Cell Counting Kit-8 (CCK-8; Epizyme, China). HUVECs were seeded into 96-well plates at a density of 5 × 10^3^ cells per well. Untreated cells served as the control group, and 50 μg/mL ox-LDL (MedChemExpress, USA) was added to the experimental group. After 24 hours of incubation, 10 μL CCK-8 solution was added to each well and incubated at 37°C for 2 hours. Optical density (OD) was measured at 450 nm using a microplate reader (Molecular Devices, USA). Cell viability (%) was calculated as (*OD*_experimental group_/*OD*_control group_) × 100%. The ox-LDL concentration and exposure duration (50 μg/mL, 24 h) were selected according to previously published studies that consistently demonstrate endothelial injury and ERS activation under similar conditions.[12]

### 2.8. Quantitative Real-Time polymerase chain reaction (qRT-PCR) for signature gene expression verification

Total RNA from each group of cells was extracted by the TRIzol reagent (Invitrogen, USA). Next, cDNA was synthesized from 1 μg of total RNA by using the RevertAid First Strand cDNA Synthesis Kit (Thermo Scientific, USA). Finally, qRT-PCR was performed on a 7500 Fast Real-Time PCR System (Applied Biosystems, USA) using a SYBR Green I Master Mix Kit (Vazyme, China). Gene expression was calculated using the 2^-ΔΔCt^ method, and β-actin was used as an internal reference. The qRT-PCR primers used in the present study are shown in Table 2.

**Table 2:** Sequences of qRT-PCR primers used in this study.

Target gene		Primer Sequence
β-actin	Forward Reverse	TCACCATGGATGATGATATCGC ATAGGAATCCTTCTGACCCATGC
TRIM25	Forward Reverse	AGCGTCCACACACAAATCCAC GGACAGGGGGAGGTTTCTTGG
CYBB	Forward Reverse	TGCCAGTCTGTCGAAATCTGC ACTCGGGCATTCACACACC
CYBA	Forward Reverse	GCATCTACCTACTGGCGGC TTGATGGTGCCTCCGATCT
PRKAA2	Forward Reverse	GTGAAGATCGGACACTACGTG CTGCCACTTTATGGCCTGTTA
MYOC	Forward Reverse	AGGCTGTTGCTGCTGTTTGT CGGTTGAGGTTGTAGGAGGA

### 2.9. Western blotting (WB) for protein expression verification

Protein expression of the five signature genes was further examined by WB in control and ox-LDL-treated HUVECs. Total cellular protein was extracted using RIPA lysis buffer supplemented with protease inhibitors and quantified using a BCA protein assay kit. Equal amounts of protein were separated by SDS-PAGE and transferred onto PVDF membranes. After blocking, the membranes were incubated overnight at 4°C with primary antibodies against TRIM25, CYBB, CYBA, MYOC, PRKAA2, and GAPDH as the loading control, followed by incubation with the corresponding secondary antibodies. Protein bands were visualized using an enhanced chemiluminescence detection system. Band intensities were quantified using ImageJ software and normalized to GAPDH. Relative protein expression in the control group was set to 1.

### 2.10. Statistical methods

Statistical analyses for the in vitro experiments were performed using GraphPad Prism 8 software (USA). Data are presented as mean ± standard deviation (SD). Technical replicates within each experiment were averaged, and the mean value of each independent experiment was used for statistical analysis. Normality of data distribution was assessed using the Shapiro–Wilk test, and homogeneity of variances was evaluated using Levene’s test. Comparisons between two groups of data were made using the independent samples t-test, and *P* < 0.05 was considered a statistically significant difference.

### 2.11. Ethics statement

This study analyzed publicly available GEO datasets and used commercially obtained HUVEC cell lines for in vitro experiments. No human participants, human specimens collected by the authors, or animal experiments were involved; therefore, institutional ethical approval and informed consent were not required.

## 3. Results

### 3.1. Identification of differentially expressed genes

**Fig 1:**
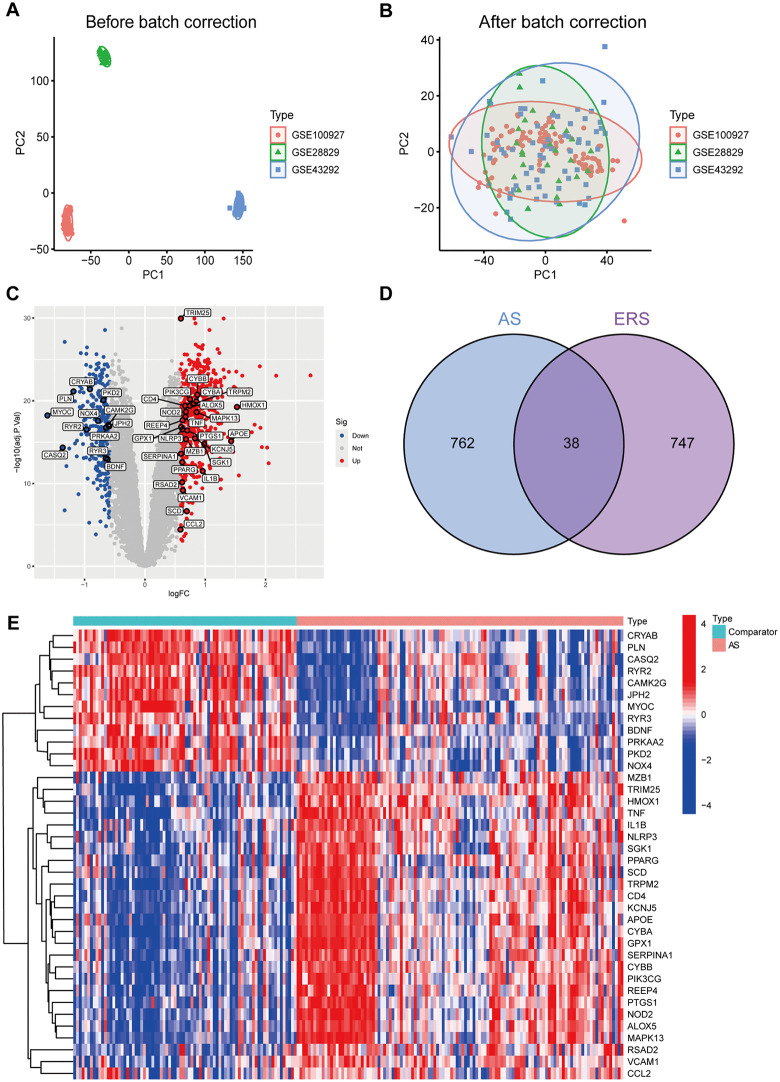
Identification of ERS-related differentially expressed genes in AS. **(A)** PCA before batch correction. **(B)** PCA after batch correction. **(C)** Volcano plot of all DEGs between AS and comparator samples, with the 38 ERS-related DEGs highlighted. **(D)** Venn diagram showing the overlap between AS-related DEGs and ERS-related genes. **(E)** Heatmap illustrating the expression patterns of the 38 ERS-related DEGs in AS and comparator samples.

After normalization, probe annotation, and batch-effect correction, the GSE28829, GSE43292, and GSE100927 datasets were integrated into a combined expression matrix (Fig 1A, B). Differential expression analysis identified 800 DEGs between the AS and comparator groups, including 516 upregulated and 284 downregulated genes. Among these, 38 genes overlapped with the ERS gene set obtained from GeneCards and MSigDB (Fig 1D). Their distribution within the overall DEG landscape was visualized in the volcano plot, in which the 38 ERS-related DEGs were specifically highlighted (Fig 1C). A heatmap further demonstrated distinct expression patterns of these 38 genes between AS and comparator samples (Fig 1E). Detailed differential expression statistics for all 38 ERS-related genes are provided in S1 Table.

### 3.2. Functional enrichment analysis of ERS-related differentially expressed genes in AS

**Fig 2:**
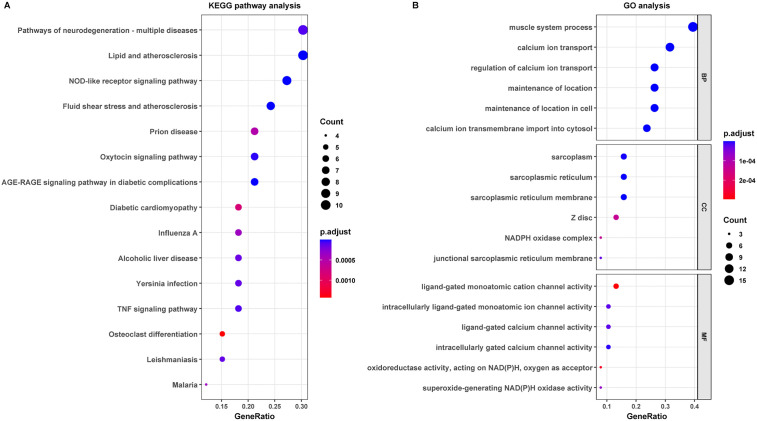
Functional enrichment analysis of the 38 ERS-related DEGs in AS. **(A)** KEGG pathway enrichment analysis. **(B)** GO enrichment analysis of biological process (BP), cellular component (CC), and molecular function (MF). The x-axis represents GeneRatio, dot size indicates gene count, and dot color represents the adjusted *P* value.

To further characterize the potential functions of the 38 ERS-related DEGs in AS, GO and KEGG enrichment analyses were performed. GO analysis indicated that these genes were primarily involved in calcium transport-related biological processes, NADPH oxidase complex-related cellular components, and oxidoreductase- and calcium channel-related molecular functions. KEGG analysis further showed significant enrichment in lipid and atherosclerosis, fluid shear stress and atherosclerosis, TNF signaling, and NOD-like receptor signaling pathways. Overall, these results suggest that ERS-related DEGs in AS are closely associated with calcium homeostasis, oxidative stress, and inflammation-related signaling (Fig 2).

### 3.3. Machine learning-based identification of candidate ERS-related signature genes in AS

**Fig 3:**
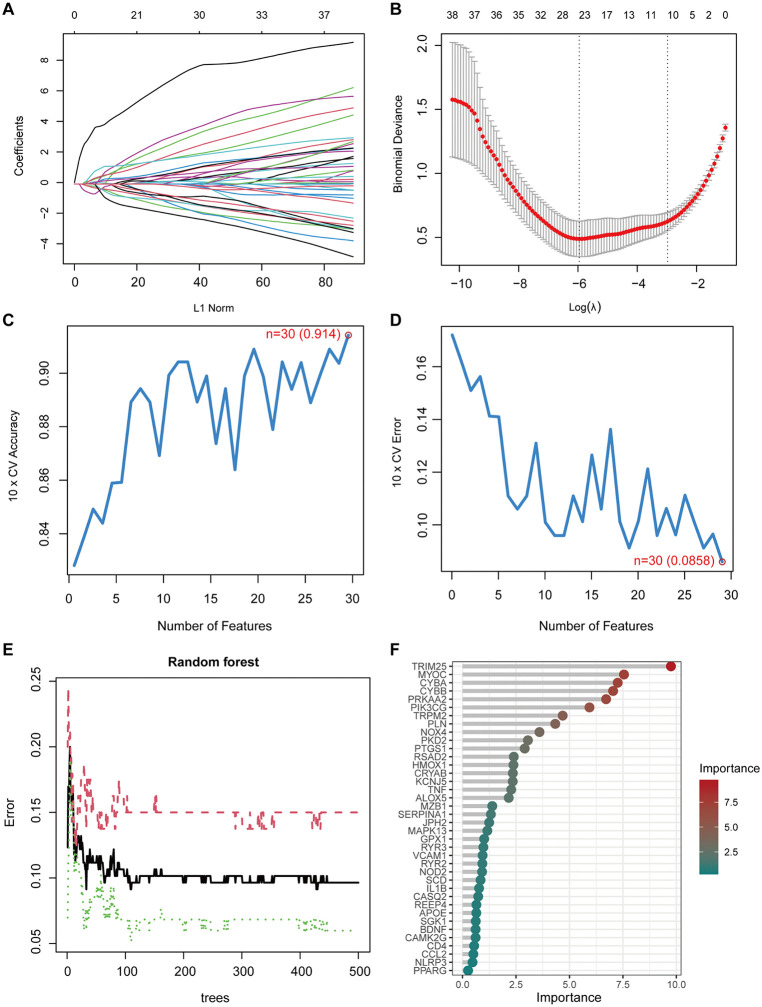
Machine learning-based identification of candidate ERS-related signature genes in AS. **(A)** LASSO coefficient profiles of 38 ERS-related differentially expressed genes. **(B)** Ten-fold cross-validation curve for LASSO regression under the binomial generalized linear model. **(C)** Cross-validation accuracy profile of SVM-RFE across different numbers of selected features. **(D)** Cross-validation error profile of SVM-RFE used to determine the optimal feature subset. **(E)** Out-of-bag (OOB) error rate of the random forest model as the number of trees increases. **(F)** Variable importance ranking of genes in the random forest model.

**Fig 4:**
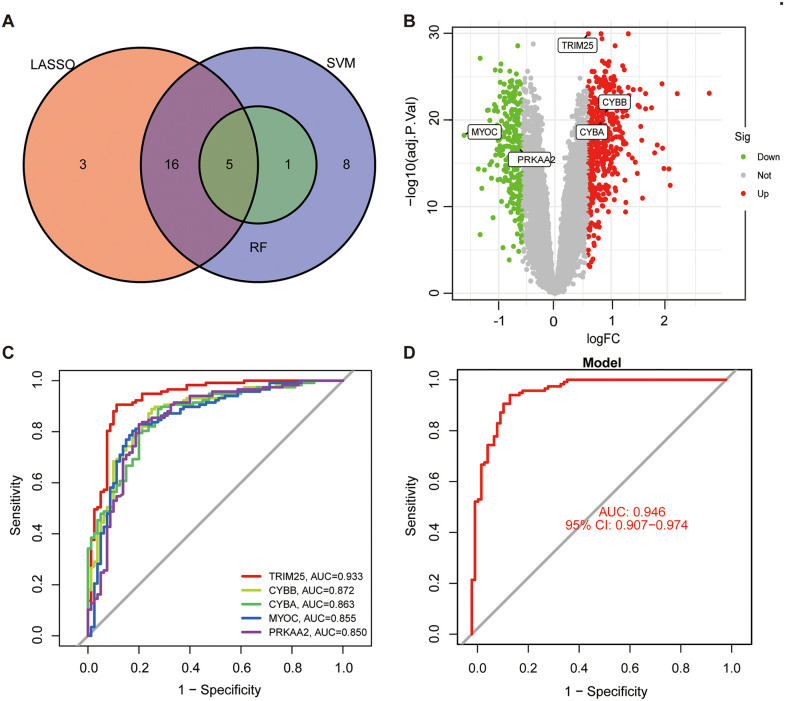
Identification and discriminatory performance of the five ERS-related signature genes in AS. **(A)** Venn diagram illustrating the overlap of genes identified by LASSO, SVM-RFE, and random forest analysis. **(B)** Differential expression summary of the five ERS-related signature genes in the integrated discovery cohort. **(C)** ROC curves of the five individual ERS-related signature genes for distinguishing AS from comparator samples in the integrated discovery cohort. **(D)** ROC curve of the five-gene logistic regression model in the integrated discovery cohort.

Among the 38 ERS-related differentially expressed genes, LASSO regression retained 24 genes with non-zero coefficients under the optimal penalty parameter determined by ten-fold cross-validation (Fig 3A, B). SVM-RFE identified an optimal subset of 30 genes contributing to discrimination between AS and comparator samples (Fig 3C, D). Random forest analysis retained 6 genes with variable importance scores greater than 5 (Fig 3E, F). Intersection of the results from the three algorithms yielded five candidate ERS-related signature genes: TRIM25, CYBB, CYBA, MYOC, and PRKAA2 (Fig 4A). In the integrated dataset, TRIM25, CYBB, and CYBA were upregulated in AS samples, whereas MYOC and PRKAA2 were downregulated (Fig 4B).

### 3.4. Discriminatory performance of the five-gene ERS-related signature in the integrated discovery cohort

ROC analysis in the integrated dataset showed that the five identified ERS-related signature genes all had good discriminatory performance for distinguishing AS from comparator samples. The AUC values of TRIM25, CYBB, CYBA, MYOC, and PRKAA2 were 0.933, 0.872, 0.863, 0.855, and 0.850, respectively (Fig 4C). Among the individual genes, TRIM25 showed the highest discriminatory performance in the integrated discovery cohort (AUC = 0.933, 95% CI: 0.892–0.964). Notably, the combined five-gene logistic regression model achieved an AUC of 0.946 (95% CI: 0.907–0.974) (Fig 4D), indicating slightly improved discrimination compared with the individual genes.

### 3.5. Experimental verification of signature gene expression at the mRNA and protein levels

**Fig 5:**
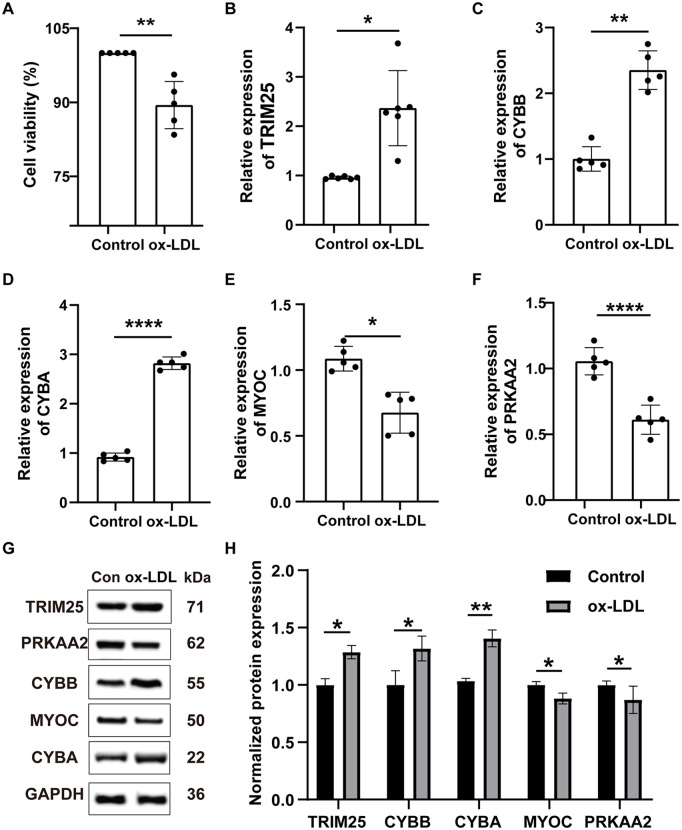
Experimental validation of the five ERS-related signature genes in an ox-LDL-induced HUVEC injury model. **(A)** CCK-8 assay showing reduced viability of HUVECs following 24-hour treatment with 50 μg/mL ox-LDL. **(B–F)** Relative mRNA expression levels of TRIM25 **(B)**, CYBB **(C)**, CYBA **(D)**, MYOC **(E)**, and PRKAA2 (**F**) in control and ox-LDL-treated HUVECs, as determined by qRT-PCR. **(G)** Representative Western blot bands of TRIM25, CYBB, PRKAA2, MYOC, CYBA, and GAPDH. **(H)** Quantitative analysis of relative protein expression. Band intensities were normalized to GAPDH and expressed relative to the control group. Data are presented as mean ± SD. In panels A–F, each dot represents one independent biological replicate (n = 5 per group). Panels G and H are based on 3 independent biological experiments. Technical replicates within each experiment were averaged before statistical analysis. Comparisons between two groups were performed using the independent samples t-test. *P < 0.05, **P < 0.01, ****P < 0.0001 compared to the control group.

To validate the bioinformatics predictions, an in vitro endothelial injury model was established in HUVECs using ox-LDL stimulation. Treatment with 50 μg/mL ox-LDL for 24 hours significantly reduced HUVECs viability compared with the untreated control group, indicating successful establishment of the cell injury model (Fig 5A). Consistent with the bioinformatics analysis, qRT-PCR showed that the mRNA expression levels of TRIM25, CYBB, and CYBA were significantly increased in ox-LDL-treated HUVECs, whereas MYOC and PRKAA2 were significantly decreased (Fig 5B-F). WB further supported these findings at the protein level. Compared with the control group, ox-LDL treatment increased the protein expression of TRIM25, CYBB, and CYBA, while decreasing the protein expression of MYOC and PRKAA2 (Fig 5G, H). Overall, the protein-level changes were directionally consistent with the transcript-level findings, providing additional experimental support for the identified ERS-related signature genes in the endothelial injury model.

### 3.6. Immune cell correlations of signature genes

**Fig 6:**
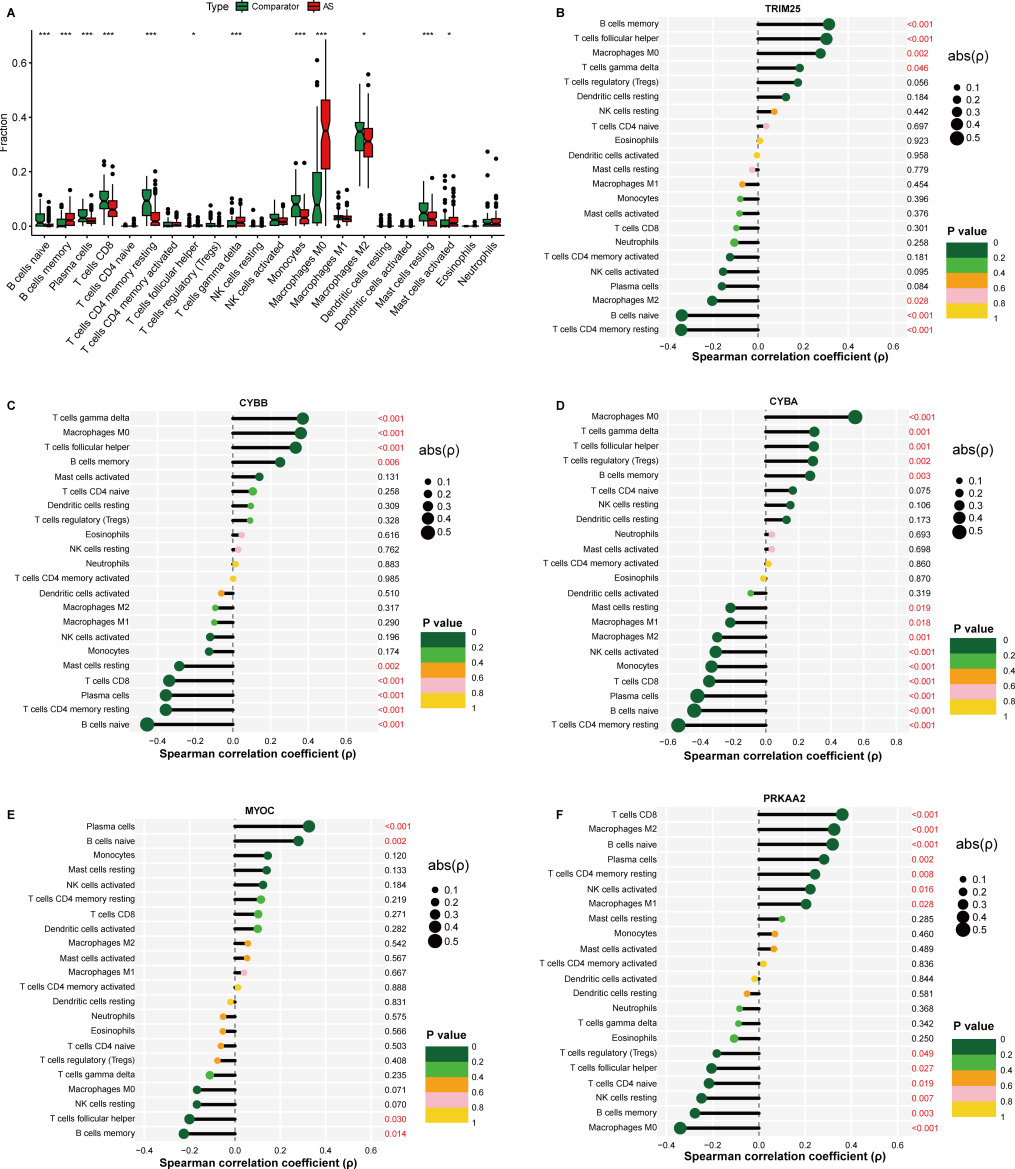
Associations between signature gene expression and immune cell infiltration. **(A)** Comparison of the CIBERSORT-estimated fractions of immune cell types between AS and comparator samples. **(B-F)** Spearman correlation analysis between the expression of TRIM25 **(B)**, CYBB **(C)**, CYBA **(D)**, MYOC **(E)**, and PRKAA2 (**F**) and the estimated fractions of 22 immune cell types across samples..

CIBERSORT-based immune infiltration analysis revealed significant differences in several immune cells in AS plaque tissues (Fig 6A). To further explore the immune-related features of the identified ERS-related signature genes, Spearman correlation analyses were performed across samples between the expression level of each signature gene and the CIBERSORT-estimated fractions of 22 immune cell types. TRIM25, CYBB, and CYBA showed multiple significant associations with selected immune cell subsets (Fig 6B-D). In contrast, MYOC did not show significant correlations with macrophages M2 or CD4 + T-cell subsets (Fig 6E), whereas PRKAA2 showed a positive correlation with the estimated CD8 + T-cell fraction (Fig 6F). Overall, these results suggest that the identified ERS-related signature genes may be associated with alterations in the immune microenvironment of AS.

### 3.7. GSEA enrichment analysis

**Fig 7:**
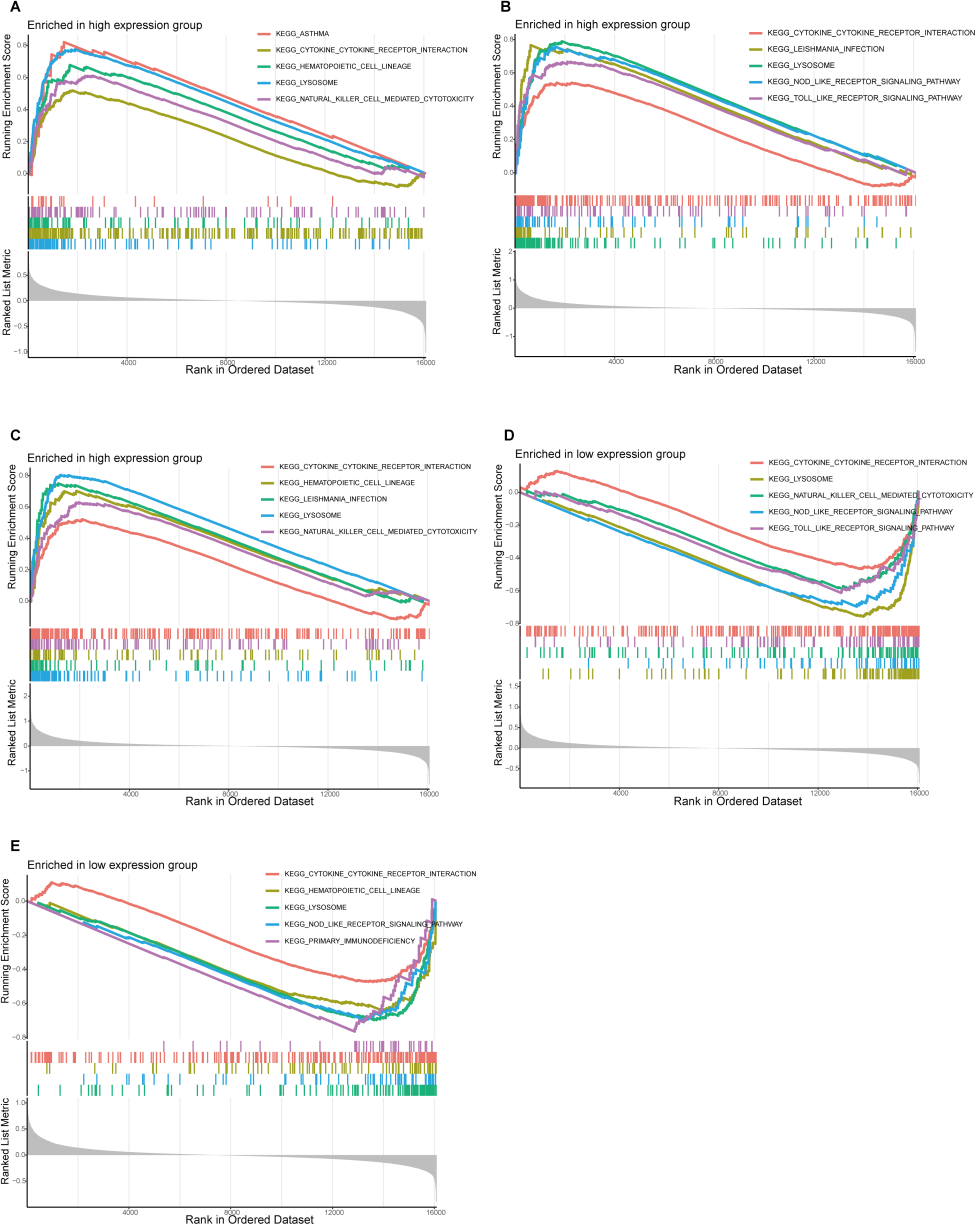
GSEA of the five ERS-related signature genes in AS. **(A)** Enriched KEGG pathways associated with high TRIM25 expression. **(B)** Enriched KEGG pathways associated with high CYBB expression. **(C)** Enriched KEGG pathways associated with high CYBA expression. **(D)** Enriched KEGG pathways associated with low MYOC expression. **(E)** Enriched KEGG pathways associated with low PRKAA2 expression.

GSEA revealed significant enrichment of immune-related KEGG pathways—such as cytokine-cytokine receptor interaction, hematopoietic cell lineage, leishmaniasis, NOD-like receptor signaling, Toll-like receptor signaling, and natural killer cell-mediated cytotoxicity—in the high-TRIM25, high-CYBB, and high-CYBA expression groups (Fig 7A-C). In contrast, low expression of MYOC and PRKAA2 was mainly enriched in natural killer cell-mediated cytotoxicity, NOD-like receptor signaling pathways, Toll-like receptor signaling pathways, hematopoietic cell lineage, lysosome, and primary immunodeficiency pathways (Fig 7D, E). Notably, there is considerable overlap in the enriched pathways, suggesting that these gene sets may coordinately regulate immune-related biological processes.

## 4. Discussion

In AS, chronic inflammation, ox-LDL deposition, and abnormal shear stress trigger ERS in endothelial cells, macrophages, and smooth muscle cells.[13] ERS promotes inflammation, disrupts lipid metabolism, and increases apoptosis, contributing to plaque formation and instability.[14] It also activates the UPR, linking metabolic stress to vascular injury.[15] In the present study, we identified ERS-related signature genes in AS through integrated bioinformatics and machine learning, providing additional insight into the molecular basis of ERS-mediated vascular injury.

By integrating multiple GEO datasets, we identified 38 ERS-related differentially expressed genes significantly associated with AS. Functional enrichment analysis indicated that these ERS-related DEGs were mainly associated with calcium homeostasis, oxidative stress, and inflammation-related pathways, supporting a close link between ERS dysregulation and vascular injury in AS. Through the application of LASSO regression, SVM-RFE, and RF algorithms, we further narrowed this set to five signature genes: TRIM25, CYBB, CYBA, MYOC, and PRKAA2. These five genes showed favorable discriminatory performance in the integrated discovery cohort. Furthermore, experimental validation in an ox-LDL-induced endothelial injury model supported the predicted expression patterns at both the mRNA and protein levels, with TRIM25, CYBB, and CYBA showing increased expression and MYOC and PRKAA2 showing decreased expression. This concordance between transcript-level and protein-level changes strengthens the biological relevance of these signature genes in AS-related endothelial stress. However, further validation in independent cohorts and more rigorous evaluation frameworks is still needed before the translational applicability of these findings can be more firmly established.

Among the identified signature genes, TRIM25 is a compelling candidate. TRIM25 (Tripartite motif-containing 25) is an E3 ubiquitin ligase involved in antiviral responses and inflammatory signaling.[16,17] Emerging evidence suggests that TRIM25 promotes M1 macrophage polarization and increases tumor necrosis factor-α (TNF-α) and interleukin-1β (IL-1β) secretion, thereby aggravating vascular inflammation and atherosclerotic plaque formation.[18] Clinically, serum TRIM25 levels correlate with the severity of coronary stenosis, and TRIM25 upregulation has also been implicated in atherosclerosis-related inflammatory signaling.[19] These findings suggest that TRIM25 may act as a pro-inflammatory mediator under ERS-related conditions in AS.

CYBB and CYBA, encoding subunits of NADPH oxidase (NOX2 complex), are key drivers of reactive oxygen species (ROS) generation.[20] They are involved in cardiovascular disease, immune responses, and other pathological processes.[21] CYBA and CYBB regulate NOX-mediated ROS production, contributing to oxidative damage and inflammatory cascades in AS. Aberrant CYBA expression enhances the activity of various NOX isoforms, thereby accelerating cardiovascular disease progression.[22] Excessive ROS accumulation can also induce ERS and activate the UPR, leading to endothelial dysfunction and apoptosis.[23] Their high expression in AS in the present study supports the idea that NOX2-mediated oxidative stress may exacerbate ERS and promotes plaque progression.

In contrast to the pro-atherogenic roles of TRIM25 and the NOX subunits, PRKAA2 represents a potentially protective factor. PRKAA2 encodes the α2 catalytic subunit of AMP-activated protein kinase (AMPKα2). In the vascular system, AMPKα2 contributes to endothelial and smooth muscle cell function. It maintains ER and calcium homeostasis by preventing sarcoplasmic-endoplasmic reticulum calcium ATPase (SERCA) oxidation, thereby reducing ERS.[24] Reduced PRKAA2 expression may exacerbate lipid accumulation and inflammatory signaling during atherogenesis.[25] Although MYOC has traditionally been studied primarily in relation to ocular physiological functions, it is also expressed in skeletal muscle and cardiac tissues.[26] This suggests that MYOC may be related to extracellular matrix regulation or vascular remodeling, although its role in AS remains less well defined and requires further investigation. Collectively, these genes reflect the molecular interactions between oxidative stress, energy metabolism, and ER homeostasis in AS. Their combined discriminatory performance further supports the view that ERS is an important integrative mechanism linking metabolic dysfunction to vascular inflammation.

Our GSEA analysis further elucidated the biological pathways most closely associated with ERS in AS. Samples with high TRIM25, CYBB, and CYBA expression were significantly enriched in immune and inflammatory pathways such as cytokine–cytokine receptor interaction, Toll-like receptor signaling, NOD-like receptor signaling, and natural killer cell-mediated cytotoxicity. These pathways reflect the strong link between ERS and innate immune activation, where excessive inflammatory signaling amplifies endothelial dysfunction. Meanwhile, low expression of MYOC and PRKAA2 was primarily associated with lysosome and primary immunodeficiency pathways, indicating impaired intracellular degradation and immune regulation under chronic ERS. Together, these results emphasize that ERS does not act in isolation but intersects with multiple immune and metabolic pathways that accelerate vascular inflammation and plaque instability.

Consistent with the GSEA results, immune infiltration analysis suggested that ERS-related gene expression was associated with remodeling of the immune microenvironment in AS. Specifically, higher TRIM25 expression was associated with increased fractions of memory B cells, T follicular helper cells, resting CD4 + T cells, and naive B cells, whereas CYBB and CYBA were more strongly associated with macrophage M0, naive B cells, and selected T-cell subsets. In contrast, MYOC showed no significant correlation with macrophages M2 or CD4 + T-cell subsets, while PRKAA2 was positively correlated with the CD8 + T-cell fraction. Although these associations do not establish causality, they support a potential link between ERS-related molecular alterations and immune microenvironment changes in AS. These immune cell populations are known to amplify inflammatory signaling and influence plaque stability.[27]

Taken together, the main implication of this study is that ERS-related molecular alterations may help identify biologically active and inflammation-associated states in AS. The ERS-related genes identified here may help to characterize high-risk disease states and provide candidate molecular clues for earlier risk stratification and more targeted preventive research. From a broader clinical and public health perspective, these findings support the need for continued efforts toward earlier detection, improved biological classification of atherosclerotic lesions, and the development of more precise intervention strategies. However, these findings should still be interpreted cautiously, as they are based mainly on public transcriptomic data and limited in vitro validation, and therefore require further longitudinal and mechanistic studies before clinical translation.

The strengths of this study lie in the integration of multiple transcriptomic datasets and machine learning algorithms to systematically identify ERS-related signature genes associated with AS, thereby enhancing the robustness and biological relevance of the findings. Additionally, immune infiltration analysis expanded insights into the inflammatory microenvironment and potential molecular mechanisms underlying AS.

## 5. Limitations

Despite the advantages of our integrated bioinformatics and machine learning approach, several limitations must be acknowledged. The analysis was based on publicly available datasets, which may still contain residual heterogeneity despite batch correction. Second, although the expression patterns of the identified genes were further examined at both the mRNA and protein levels in an ox-LDL-induced HUVEC injury model, the experimental validation remained limited to an in vitro endothelial context and lacked direct verification in human atherosclerotic plaque tissues or animal models. In addition, integrating additional multi-omics approaches, such as proteomics, metabolomics, and single-cell sequencing, may provide a more comprehensive understanding of the molecular networks associated with ERS in AS.

## 6. Conclusion

In conclusion, this study systematically identified five ERS-related signature genes, including TRIM25, CYBB, CYBA, MYOC, and PRKAA2, that were significantly associated with AS through integrated bioinformatics and machine learning approaches. Their discriminatory performance was supported in the integrated discovery cohort, and their expression trends were further validated at both the mRNA and protein levels in an ox-LDL-induced endothelial injury model. Immune infiltration and pathway analyses suggested that these genes are linked to oxidative stress, inflammatory signaling, and immune-related changes relevant to AS pathogenesis. These findings provide additional evidence for ERS-associated molecular alterations in AS and identify candidate ERS-related markers that warrant further tissue-level and mechanistic validation. In practical terms, these findings suggest that stress- and inflammation-related molecular changes in atherosclerosis may, in the future, contribute to earlier recognition of biologically active disease and support the development of more targeted prevention strategies.

## 7. Future Directions

Future studies should further evaluate these signature genes in well-characterized cohorts with more consistent clinical definitions and lesion contexts. In particular, longitudinal studies are needed to clarify whether these genes are associated with disease progression or dynamic changes during atherosclerotic development. In addition, mechanistic studies at the cellular and molecular levels are required to determine how these genes contribute to ERS-related vascular injury and inflammatory regulation in AS. The integration of additional approaches, such as proteomics, metabolomics, and single-cell sequencing, may also provide a more comprehensive understanding of ERS-related molecular networks in AS.

## Supporting information

S1 Table Differential expression statistics of the 38 endoplasmic reticulum stress-related genes in the integrated atherosclerosis cohort. (XLSX)

S1 Data Raw data supporting Fig 6. (XLSX)

S1 Raw images Original Western blot images. (TIF)
