# Supplementary figures and images for: Screening and comprehensive analysis of endoplasmic reticulum stress-related biomarkers in atherosclerosis
Source: PLoS One. 2026 Jun 1;21(6):e0350047. doi: 10.1371/journal.pone.0350047 (PMC13225432; doi:10.1371/journal.pone.0350047)

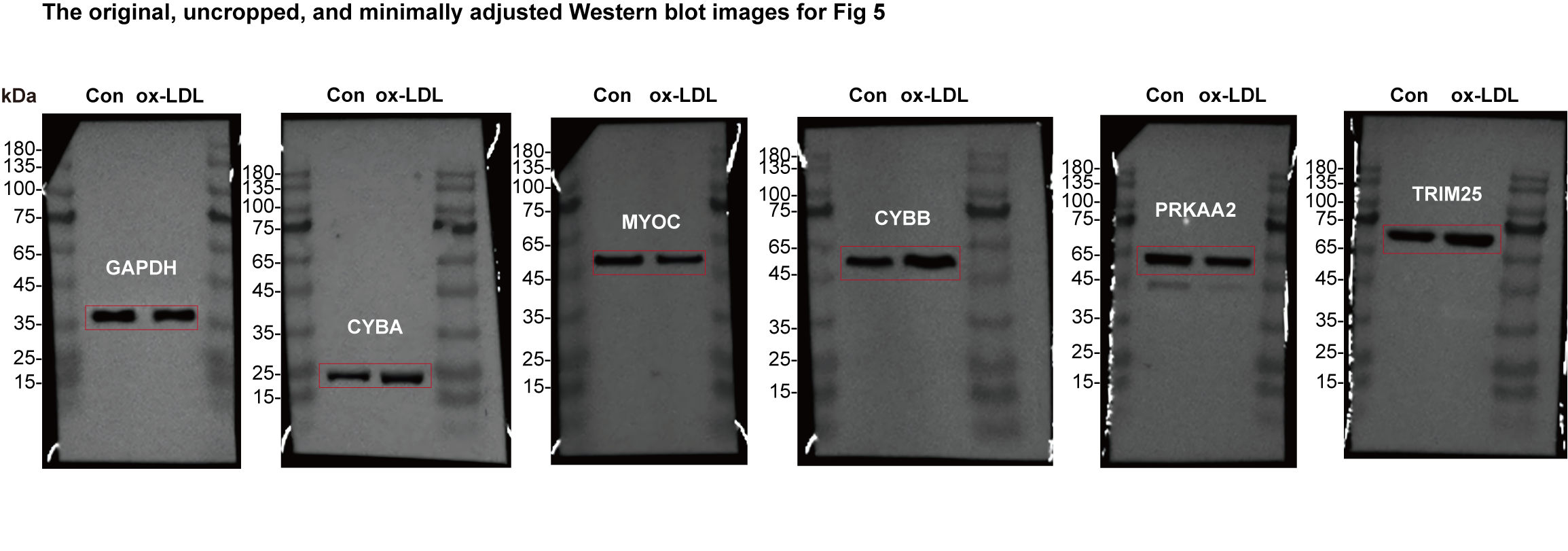

Supplement: S1 Raw images — (TIF) [file pone.0350047.s003.tif]
